# Predictors of Postherpetic Neuralgia: A Prospective Study From Eastern Nepal

**DOI:** 10.1111/jocd.70136

**Published:** 2025-04-14

**Authors:** Suchana Marahatta, Shivendra Kumar Jha, Ashish Ghimire

**Affiliations:** ^1^ Department of Dermatology & Venereology B. P. Koirala Institute of Health Sciences Dharan Sunsari Nepal; ^2^ Department of Dermatology and Venerology Sukraraj Tropical and Infectious Diseases Hospital (STIDH) Teku Kathmandu Nepal; ^3^ Department of Anaesthesiology and Critical Care B. P. Koirala Institute of Health Sciences Dharan Sunsari Nepal

**Keywords:** dermatome, herpes zoster, postherpetic neuralgia, trigeminal neuralgia, varicella‐zoster virus

## Abstract

**Background:**

Herpes zoster (HZ) is a viral infection with severe acute and chronic pain. This study was aimed at determining the incidence rate of postherpetic neuralgia (PHN). The objectives of the study were to understand the epidemiology of HZ and to identify the risk factors for PHN at a referral hospital in eastern Nepal.

**Materials and Methods:**

A total of 82 patients with HZ presenting within 72 h of skin eruption with a pain rating of 40 or above on a visual analog scale, with an age ≤ 75 years and those willing to participate were included in the study. However, those who had already started antiviral medicine or analgesics were excluded. All baseline details were noted and the participants were followed for 16 weeks at monthly intervals to assess the pain status and development of PHN. The epidemiological parameters and the predictors of PHN were predicted using the chi‐square test and logistic regression analysis.

**Results:**

The mean age of the participants was 47 ± 18.77 years. The majority (32.9%) of the participants belonged to 61–70 years. Thoracic dermatome was most frequently (56.1%) involved. At the 16‐week follow‐up, of the 77 patients who completed the study, 14 had persistent pain; hence, the incidence of PHN was 18.2%. Advancing age (> 50 years) (adjusted odds ratio 2.95, 95% confidence interval (CI) 1.33–6.53); diabetes (OR 13.09, 95% CI 1.40–122.24); and prolonged prodromal pain (OR 12.01, 95% CI 1.40–102.77) were the most important risk factors for PHN.

**Conclusion:**

In our study, the prevalence of PHN was quite high (18.2%) despite timely antiviral treatment and regular follow‐up. An age of more than 50 years, prolonged prodromal pain, and diabetes were the most significant predictors of PHN. Hence, early intervention and frequent follow‐up of those individuals are recommended.

## 1 Introduction

Herpes zoster (HZ), also known as “shingles” is a common viral infection caused by the reactivation of varicella‐zoster virus present in the latent form in the sensory nerve ganglia. Its average annual incidence is approximately 3.6 in the United States and five in Asia‐pacific per 1000 population [1, 2]. However, it is more prevalent in our country (seven per 1000 populations) as reported in a study from central Nepal [3]. However, there are no published data available from other parts. HZ is characterized by unilateral, dermatomal, painful self‐limiting vesicular skin eruption. It causes both acute and chronic distressing dermatomal pain. The pain of HZ is so notorious that it affects physical, social, and emotional well‐being.

The most bothering complications of HZ are postherpetic neuralgia (PHN), which is defined as the pain persisting beyond 4 months from the onset of HZ prodrome [4, 5]. The incidence rate varies from 12.8% to 29.8% in different reports [6, 7]. Factors like advancing age, greater acute pain intensity, a greater number of rashes, and longer duration of prodromal pain are some risk factors for PHN [8, 9]. However, they have enrolled patients even after many days of rash onset with variable baseline pain intensity. There is a paucity of data throughout the world and no data from Nepal to date. Knowing the epidemiology of HZ and predictors of PHN, we can initiate timely prevention strategies. The aim of this study was to determine the incidence rate of PHN. The objectives of the study were to understand the epidemiology of HZ and to identify the risk factors for PHN at a referral hospital in eastern Nepal.

## 2 Materials and Methods

We conducted this study in a tertiary care referral hospital of eastern Nepal. The study included a total of 82 patients with HZ from August 2018 to July 2019. Patients with HZ with ages 18–75 years, presenting with a rash onset within 72 h, having a pain rating of 40 or above on a 100 mm visual analog scale (VAS), and giving informed written consent were included in this study. However, those who took oral analgesics within 6 h of the screening visit, on antiviral medicine, had unstable medical conditions, malignancy, severe comorbidities, and psychiatric illness, or were pregnant and breastfeeding were excluded from the study.

The sociodemographic characteristics, detail relevant history like duration of prodromal symptoms, duration of skin eruption, severity of the pain, associated symptoms, and examination findings were recorded in the preset pro‐forma. The VAS was used to assess the severity of pain, which comprises 0–100 mm horizontal lines, where “0” stands for no pain at all and “100” stands for “worst pain” [10]. All patients were prescribed Acyclovir 800 mg five times a day for 7 days. Patients were followed up at 16 weeks from the baseline visit to assess the pain status and to find out the incidence of PHN.

### 2.1 Case Definition of PHN

The pain persisting beyond 4 months from the onset of HZ prodrome [4].

### 2.2 Ethical Clearance

Ethics approval was obtained from the institutional research committee with reference number (IRC/1225/018). This study is part of original research entitled “Efficacy of pregabalin for the treatment of acute herpetic neuralgia and the prevention of postherpetic neuralgia‐a randomized controlled trial.”

### 2.3 Statistical Analysis

Data were entered in Microsoft Excel 2010 and converted into Statistical Package for Social Science (SPSS) version 11.5. For descriptive statistics, percentage, frequency, proportion, mean, median, mode, standard deviation, and IQR were used. Similarly, for inferential statistics, we applied the chi‐square test. Logistic regression analysis was conducted to find the predictors of PHN at 95% CI, where a *p* value < 0.05 was considered significant.

## 3 Results

### 3.1 Clinico‐Epidemiological Profiles of HZ

**FIGURE 1:**
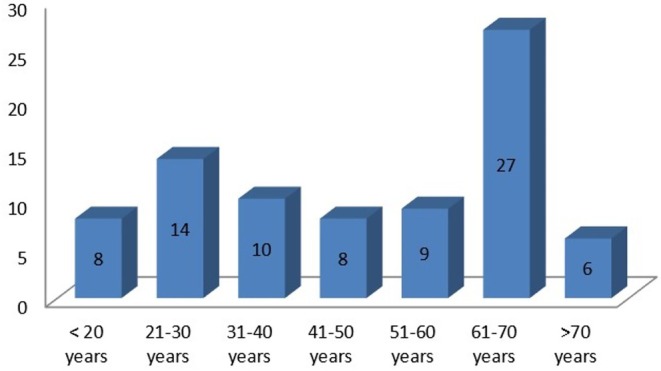
Age distribution of patients (*x*‐axis: age in years, *y*‐axis: number of patients).

A total of 82 patients were enrolled in this study. The mean age of the participants was 47 ± 18.77 years. The majority (32.9%) of the participants belonged to the age group of 61–70 years. However, we also have a significant number of participants (9.8%) at age ≤ 20 years (Figure 1). Male and female were almost equal with a 1:1 ratio. Most of the participants, 25 (30.5%) were housewives, followed by farmers 19 (23.2%), government service holders 14 (17.1%), students 13 (15.9%), and business persons 11 (13.4%). Twenty (24.4%) participants were illiterate.

Mean duration of prodromal symptoms was 3.79 ± 2.29 days. Malaise was present in half of the participants (Table 1). Intermittent type of pain, 45 (54.9%) was slightly more than the persistent, 37 (45.1%). Fifty‐nine (72.0%) participants pointed to some possible causes of reactivation of the disease; the most common being physical stress in 26 (40.2%) patients (Table 1).

**TABLE 1:** History of the patient (cause of reactivation and associated symptoms).

Characteristics	Category	No. (*n* = 84)	Percentage
Causes of reactivation	*No*	23	28.0
	*Yes*	59	72.0
	Infection	20	24.4
	Physical stress	26	40.2
	Mental stress	17	20.7
	Diabetes mellitus	7	8.5
	Systemic steroid intake	1	1.7
Associated symptoms	Headache	29	35.4
	Itching	27	32.9
	Malaise	26	31.7
	Fever	15	18.3

**FIGURE 2:**
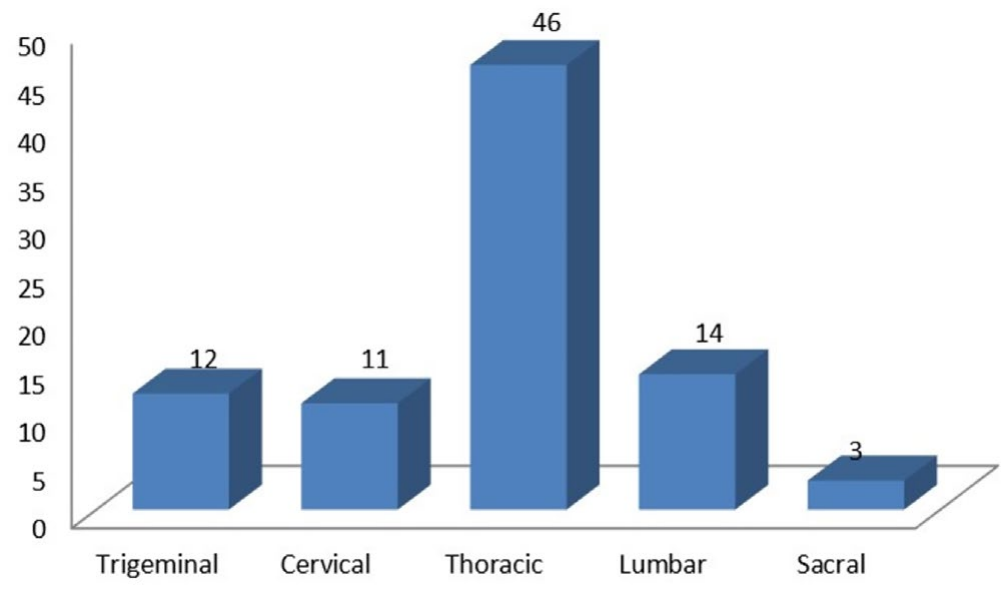
Site of involvement of herpes zoster (*x*‐axis: dermatome involved, *y*‐axis: number patients).

The onset of pain was together with skin eruption in 49 (59.75%) and before skin eruption in 33 (40.25%) participants. Thoracic dermatome was the most frequently (56.1%) involved site, followed by lumbar, 14 (17.1%), and trigeminal, 12 (14.6%) (Figure 2). Out of these, 12 patients had trigeminal dermatome involvement, 10 had ophthalmic nerve involvement, and two had maxillary nerve involvement. However, mandibular nerve involvement was not found among our participants.

### 3.2 Postherpetic Neuralgia and Its Predictors

Out of 82 patients, only 77 completed the follow‐up visit at 16 weeks. Out of 77 patients, 14 had persistent pain. Hence, the incidence of PHN was 14/77 (18.18%). The significant risk factors for PHN on the bivariate analysis (Table 2) were advancing age (> 50 years), associated headache, pain before skin eruption, diabetes, and trigeminal dermatome involvement. On logistic regression analysis, age > 50 years, prodromal pain, and diabetes were found to be significant risk factors for the PHN (Table 3).

**TABLE 2:** Pain status at 16‐week follow‐up.

Characters		Pain present	Pain absent	*p*
Age	< 25 year	0 (0.0%)	15 (19.5%)	< 0.001
	25–50 year	0 (0.0%)	23 (29.9%)	
	> 50 year	14 (18.2%)	25 (32.5%)	
Headache	Yes	10 (13.0%)	17 (22.1%)	0.004
	No	4 (5.2%)	46 (59.7%)	
Malaise	Yes	11 (14.3%)	15 (19.5%)	0.002
	No	3 (3.9%)	48 (62.3%)	
Prodromal pain	Yes	10 (13.0%)	22 (28.6%)	0.012
	No	4 (5.2%)	41 (53.2%)	
Diabetes	Yes	5 (6.5%)	1 (1.3%)	< 0.001
	No	9 (11.7%)	62 (80.5%)	
Trigeminal dermatome	Yes	6 (7.8%)	6 (7.8%)	0.002
	No	8 (10.4%)	57 (74.0%)	

**TABLE 3:** Odds ratios (OR) for the risk of PHN.

Crude OR				Adjusted OR		
Baseline variables	OR	95% CI	*p*	OR	95% CI	*p*
Age > 50 years	12.92	2.74–60.85	< 0.001	2.95	1.33–6.53	0.008
Headache	4.64	1.57–13.74	0.004	—	—	—
Malaise	11.73	1.89–47.68	0.002	—	—	—
Prodromal pain	4.66	1.31–16.60	< 0.001	12.01	1.40–102.77	0.023
Diabetes	28.62	3.17–258.21	< 0.001	13.09	1.40–122.24	0.024
Trigeminal dermatome	4.39	1.22–15.80	0.002	—	—	—

Abbreviations: CI, confidence intervals; PHN, postherpetic neuralgia.

## 4 Discussion

In our study, the mean age of the participants was 47.55 ± 18.77 years. The majority (32.9%) of the participants belonged to 61–70 years. Thoracic dermatome was most frequently (56.1%) involved. The incidence of PHN was 18.18%. The most important predictors of PHN are advancing age (> 50 years), diabetes, prodromal pain before skin eruption, headache, malaise, and trigeminal dermatome involvement.

As reported in many previous studies, our study also confirmed that HZ was more common in the elderly [6, 11, 12, 13, 14]. More than half (51.3%) of our participants were > 50 years old. This would have been even higher if we had not excluded patients of age > 75 years because of follow‐up difficulties. However, contrary to these previous studies, we also found a significant number of patients with HZ in their twenties. More than one‐fourth (27%) of participants in our study were < 30 years. The finding was comparable to another report from central Nepal [3]. This could be explained as our national vaccination protocol does not include varicella vaccine in the list of mandatory ones.

Unlike other studies where female preponderance was seen, we did not find any gender predilection in ours. We had an almost equal number of males (43, 52.4%) and females (39, 47.6%) [3, 12, 14]. In our study, almost three‐fourths (72%) of the patients pointed to some causes for the reactivation of HZ. Among all, physical stress was the most frequently (40%) reported cause. Similarly, mental stress and diabetes were pointed out by 21% and 8.5% of participants, respectively. This finding is compatible with a recent meta‐analysis [13].

Thoracic dermatome was the most commonly (56.1%) affected dermatome followed by lumbar, 14 (17.1%) and trigeminal, 12 (14.6%) (Figure 2). Out of 12 patients with trigeminal dermatome involvement, 10 had ophthalmic and two had maxillary nerve involvement. However, mandibular dermatome was not involved in our cases. Similar to our findings, the thoracic dermatome was the most commonly affected dermatome even in other studies from central Nepal. However, unlike these studies where lumbar dermatome was not so commonly affected, in our study, it was the second most common dermatome. But, trigeminal dermatome involvement is comparable in all three studies [3, 15]. The frequency of different dermatomal involvement in terms of percentage (Thoracic>Lumber>Trigeminal>Cervical>Sacral) was comparable to another study from India [16].

### 4.1 Postherpetic Neuralgia

The incidence of PHN was (18.18%) in our study. There is a huge difference in the incidence of PHN in different countries. It can vary from 5% to 30% [17]. One recent report from China reported that 29.8% of patients with HZ developed PHN, which is significantly higher than our case [6]. The actual incidence of PHN in our population could also be more than what we have reported, as we had excluded patients with age > 75 years and serious systemic comorbidities to avoid follow‐up difficulties, as we had a longer follow‐up period of 4 months. Another possible reason for the lower incidence of PHN in our study is that we have considered PHN for persistent pain 4 months after the appearance of a rash [4]. However, in this study, they have defined PHN as persisting pain for > 1 month following the healing of the HZ rash. Also, we have tried to match the subjects by enrolling patients with pain scores ≥ 40 mm on VAS. Moreover, we recruited only those patients who presented within 72 h of the rash eruption, as a delay in the administration of treatment might have a direct impact on both acute and chronic pain [18].

### 4.2 Predictors of Postherpetic Neuralgia

We found that advanced age (> 50 years), prolonging prodromal pain before skin eruption, trigeminal dermatome involvement, associated diabetes, and associated prodromal symptoms like headache and malaise were significant risk factors for the PHN on bivariate analysis. Age > 50 years (adjusted odds ratio [OR] 2.95, 95% confidence interval [CI] 1.33–6.53), prodromal pain (adjusted OR 12.01, 95% CI 1.40–102.77) and diabetes (adjusted OR 13.09, 95% CI 1.40–122.24) were significant risk factors on logistic regression analysis as well (Table 3).

The significant predictors of PHN like advancing age, longer duration of prodromal pain were comparable to past reports. Recent meta‐analysis reported that each 10‐year increment in age, periocular lesions, larger areas of skin lesions, delay in initial treatment, prodromal pain, and pain severity in acute phase are the most important risk factors for PHN [14]. However, unlike these studies, we did not find the severity of baseline pain as a significant risk factor for PHN in our study [5, 8, 9, 12, 19]. This could be because we included only those patients with significant baseline pain score i.e. ≥ 40 mm on 100 mm VAS. Hence, all our participants had moderate to severe pain at the time of presentation.

We also found that trigeminal dermatome involvement (OR 4.39, 95% CI: 1.22–15.80, *p* = 0.002) is an important predictor for PHN. This finding is comparable to the previous study [19]. Opstelten et al. (2007) and Ding et al. (2024) reported similar findings. They reported that ophthalmic localization is one of the important predictors of PHN, with an odds ratio of 2.3 (95% CI 1.0–4.6) and 1.96 (95% CI 1.75–2.20), respectively [9, 14]. This could be explained as the trigeminal nerve has a complex neuronal course with a relatively superficial location. We also found that associated constitutional symptoms like headache (OR 4.64, 95% CI: 1.57–13.74, *p* = 0.004) and malaise (OR 11.73, 95% CI: 1.89–47.68, *p* = 0.002) are significant predictors of PHN. This is an unexpected new finding in our study, which has not been reported yet in other published studies. In a viral infection, the headache and malaise could be because of the release of inflammatory markers, and those inflammatory markers might cause more nerve injury and ganglionitis leading to PHN. As headache and malaise are commonly expected problems in any viral infection, both patients and treating doctors may not give much attention to inquire details about these symptoms. So, it is likely that we might have missed these factors as PHN predictors in past studies.

As reported previously by many studies, we also found that associated comorbidities like diabetes are significant risk factors (adjusted OR 13.09, 95% CI: 1.40–122.24, *p* < 0.024) for PHN. However, as we had excluded individuals with malignancy, HIV, severe systemic diseases, and very elderly patients (> 75 years) because of follow‐up difficulties, these factors could not be assessed for the risk of PHN [5, 14, 20].

To conclude, although HZ is common among the elderly, it is becoming increasingly common in younger age groups as well. Further studies are necessary to identify this change in epidemiology. Physical and mental stress are common provoking factors for HZ. The incidence of PHN is 18.18% in our study. However, if we consider elderly and debilitated patients, it would be even higher in our part of the world. Out of the evaluated factors, advancing age (> 50 years), prolonged prodromal pain, associated diabetes, and history of prodromal symptoms like headache and malaise are important predictors of PHN in our study. Therefore, we must consider these risk factors to outrace individuals at higher risk of PHN, and early intervention must be planned to prevent PHN in these individuals with frequent follow‐up, optimized treatment, and counseling.

### 4.3 Limitations of the Study

Single centered study with less number of sample size is one of the major limitations of the study. The confidence intervals were wide for many variables of PHN. A larger sample size and multicenter would overcome this limitation. As we could not include very old patients and those with severe comorbidities, we might have missed some of the important risk factors.

## Conflicts of Interest

The authors declare no conflicts of interest.

## Data Availability

The data that support the findings of this study are available upon request from the corresponding author. The data are not publicly available due to privacy or ethical restrictions.
